# Comparative effects of synthetic and natural hydrogels enriched with fertilizer on poppy yield and soil health in drought-prone conditions

**DOI:** 10.1038/s41598-025-28213-0

**Published:** 2025-12-29

**Authors:** Tomáš Kriška, Jiří Antošovský, Martin Brtnický, Jiří Kučerík, Jiří Holátko, Josef Jančář, Petr Škarpa

**Affiliations:** 1 Department of Agrochemistry, Soil Science, Microbiology and Plant Nutrition, Faculty of AgriSciences, Mendel University in Brno, Zemědělská 1, Brno, 61300 Czech Republic; 2 Institute of Materials Science, Faculty of Chemistry, Brno University of Technology, Purkyňova 118, Brno, 61200 Czech Republic

**Keywords:** Hydrogel, Culinary poppy, Yield, Soil health, Profitability of fertilization, Ecology, Ecology, Environmental sciences, Plant sciences

## Abstract

The negative effects of agricultural drought are particularly pronounced in spring crops, which are generally less tolerant to dry periods. One such crop frequently affected by drought is poppy (*Papaver somniferum* L.). Hydrogels enriched with fertilizer represent a promising technology to enhance water availability for plants and improve nutrient uptake from applied fertilizers. The aim of this research was to compare the effects of standard fertilizer (NPKS), a natural-based (NHA) hydrogel, a synthetic hydrogel (SAP), and both hydrogels enriched with fertilizer (NHA-NPKS and SAP-NPKS) on culinary poppy yield, the agronomic efficiency of N fertilization (AE_N_) and soil microbial activity. Each treatment was applied in two dosages (I and II). Results from a three-year field experiment showed that the application of SAP-NPKS at the lower dose (I) significantly increased seed yield. The highest AE_N_ was also observed in the SAP-NPKS I treatment. The highest seed yield overall was achieved with the higher dose of the natural-based hydrogel enriched with fertilizer (NHA-NPKS II). Furthermore, the use of NHA and NHA-NPKS significantly increased soil microbial activity. These findings suggest that fertilizer-enriched natural-based hydrogels are a promising approach for improving soil moisture retention and nutrient availability, particularly under drought conditions in poppy cultivation.

**Supplementary Information:**

The online version contains supplementary material available at 10.1038/s41598-025-28213-0.

## Introduction

 Climate change is increasingly altering environmental conditions, directly affecting the cultivation of field crops. The rise in temperature and shifts in precipitation patterns have led to a higher incidence of droughts^1^, which adversely impact crop production and ecosystem services. Yield losses due to drought stress depend on the timing, duration and severity of the drought^2^. The negative effects of agricultural drought are particularly pronounced in spring-sown crops, which are generally less tolerant to water deficit. One such a crop frequently affected by drought is poppy (*Papaver somniferum* L.). This oilseed crop is especially vulnerable to unfavourable weather conditions early in the growing season, particularly during germination and emergence^3^. Efficient soil moisture management is therefore crucial for successful poppy cultivation.

The use of synthetic superabsorbent polymers (SAPs) to enhance the water retention capacity of topsoil has been practiced for over two decades^4^. These polymers can absorb and subsequently release many times their weight in water to support plant growth over time^5^. As a result, they increase the soil´s water holding capacity^6^ and help to mitigate plant drought stress^7^. In addition, hydrogels reduce erosion and nutrients leaching during heavy rainfall, improve soil structure and prevent compaction^8^. Despite the efforts to use hydrogels based on natural polymers or their organic-inorganic hybrids, yet, the mostly used hydrogels are of synthetic origin (abbreviated here as SAPs), such as acrylate and acrylamide monomers^9^. The popularity of SAPs is caused particularly due to their low production demands and costs^10^. The beneficial properties of SAPs are well-documented, but their introduction into soil systems may also pose several risks. A major concern is the persistence of polyacrylic acid due to its extremely low biodegradation rates in soil (e.g., 0.2–0.5% over year)^11,12^.

In accordance to Commission Delegated Regulation (EU) 2024/2770^13^, continuous use of SAPs for water retention improvement in soil is conditional. It is required an ultimate degradation of at least 90% of SAPs (relative to the reference material) within 48 months plus the indicated functionality period. Second option includes mineralization of at least 90% measured by evolved CO_2_, within the same timeframe (according to the test method EN ISO 17556:2019^14^). Nevertheless, due to the low biodegradability of SAPs, current research efforts turned to developing biodegradable, environment-friendly alternatives^15–19^.

Indeed, over the last decade, natural-based hydrogel alternatives (NHAs) have shown promise as eco- friendly and cost-effective substitutes. A specific group, inorganic hydrogels, appeared to be limited by low swelling capacity and adverse effects on soil fertility^20,21^. This shifted the attention to NHAs derived from biopolymers such as polysaccharides^22^ (e.g. cellulose, starch, chitosan or various gums) or proteins^7^ (e.g. gelatine).

In addition to water availability, adequate nutrient supply is critical for optimal plant growth. For poppy, the most important nutrients include nitrogen (N), phosphorus (P), potassium (K), and sulphur (S). Nitrogen is essential for synthesis of amino acids, nucleic acids, enzymes and chlorophyll, playing a key role in biomass production^23,24^. Phosphorus is involved in synthesis of nucleic acids and phospholipids, respiration, glycolysis, lipid metabolism and energy transfer^25^. Potassium contributes to ion homeostasis, osmoregulation, enzyme activation, and membrane protein transport^26^. Sulphur is critical for the synthesis of sulphur-containing amino acids (e.g. cysteine, methionine) and certain vitamins^27^ and it supports vegetative growth^28^.

These macronutrients are usually supplied through mineral fertilizers. However, a significant portion is often lost through leaching into deeper soil layers, immobilization in soil, volatilization, runoff^29^, thereby reducing nutrient-use efficiency. As a result, only about 45% of applied nitrogen fertilizer is typically utilized by crops^30^. Therefore, improving synchronization between nutrient availability and crop demand is crucial for both economic and environmental sustainability.

Sustainable agriculture aims to introduce innovative plant nutrition systems that enhance fertilizer efficiency. One such strategy involves the application of NHA-based hydrogels in combination with mineral fertilizers to simultaneously improve soil water retention and nutrient availability. These bio-based polymers, when combined with conventional mineral fertilizers, can potentially hold substantial quantities of water and nutrients, releasing them in sync with plant demand. In case of SAPs their capacity to serve as carriers and regulators of nutrient release, reducing nutrient losses while sustaining plant growth have already been well-documented^31,32^. In contrast, broader adoption of NHAs is still limited by gaps in understanding the mechanism of nutrient release, the impacts on soil physical, chemical and biological properties as well as on plant root development^33^. Nonetheless, several studies have already indicated that NHAs can bind nutrients and release them in a controlled manner^22^. Furthermore, multicomponent NHAs have been found to slow nitrogen release, enhance soil moisture retention, and partially mitigate the environmental risks associated with SAPs^18,34^.

The aim of this study was to evaluate the multi-year effect of SAPs and NHAs enriched with fertilizer (NPKS) on the seed yield of culinary poppy. To the best of our knowledge, the impact of specific nutrient-enriched hydrogels on culinary poppy has not yet been investigated in this context. The main hypothesis was that fertilizer-enriched natural hydrogels would achieve equal or superior yield outcomes compared to synthetic SAPs. To test this hypothesis, a three-year field experiment (2022–2024) was conducted under real agricultural conditions.

## Materials and methods

### Experimental locality and climate-soil conditions

**Fig. 1:**
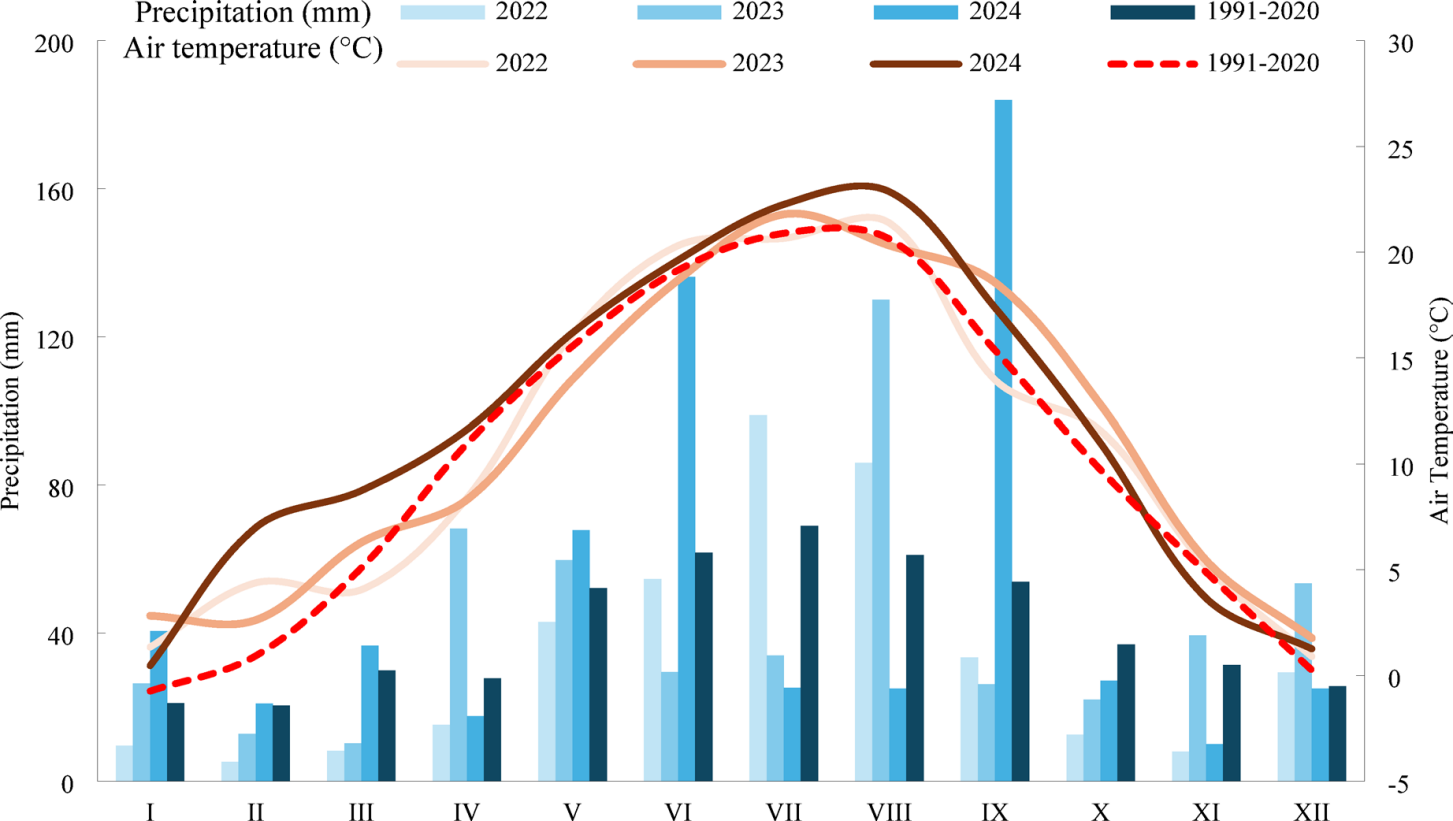
Weather conditions during the field experiment (2022–2024).

The effect of fertilizer-enriched natural and synthetic hydrogels on poppy yield was evaluated in a three-year (2022–2024) small-plot field experiment. The trial was conducted at the Žabčice experimental station in South Moravia, Czech Republic (49°1′18.658″ N, 16°36′56.003″ E), at an elevation 184 m above sea level. The site is characterized by mild, wet winters and warm, somewhat dry summers, with an average annual temperature 10.1 °C and annual precipitation of approximately 490 mm. According to the Köppen climate classification, the region falls within the “Cfb” category (temperate oceanic climate). The total precipitation and average air temperature during the experimental growing seasons were 121 mm/11.8 °C (2022), 159 mm/11.0 °C (2023), and 205 mm/14.4 °C (2024). Average monthly temperatures and precipitations during the experimental period, along with the 1991–2020 climatic norm, are presented in Fig. 1.

The experiment was conducted on a single field (240 m × 150 m), which was divided into three experimental Sect. (80 m × 150 m). Each year, poppy was grown on a different section, always following a spring barley pre-crop. Key physicochemical properties of the topsoil (0–30 cm) over the three years are presented in Table 1.

**Table 1:** Physicochemical properties of the experimental soil (0–30 cm depth).

Soil Parameter	Year		
	2022	2023	2024
Clay (%)	38.0	40.2	41.9
Silt (%)	46.3	45.1	44.1
Sand (%)	15.7	14.7	14.0
pH/CaCl_2_	6.9	6.5	6.6
P (Mehlich 3, mg/kg)	123	115	121
K (Mehlich 3, mg/kg)	244	166	267
Ca (Mehlich 3, mg/kg)	3681	1967	3015
Mg (Mehlich 3, mg/kg)	172	189	194
N-NH_4_ (K_2_SO_4_, mg/kg)	2.3	1.2	1.6
N-NO_3_ (K_2_SO_4_, mg/kg)	8.1	11.0	17.3

The clay/silt/sand content was determined according to Gee and Bauder^35^, soil acidity (pH/CaCl_2_) and nutrient content were determined according to Zbíral et at^36^..

### Experimental design and treatments

The objective was to evaluate the effect of two types of hydrogels (synthetic and natural) enriched with nutrients (N, P, K, S) on poppy (*Papaver somniferum* L.) yield. The natural hydrogel (NHA) was prepared from potato starch (AGRANA Beteiligungs-AG, Konstanz, Germany), glycerol (PENTA, Ltd., Prague, Czechia), clinoptilolite zeolite (particle size 2 mm; Rosteto, Jindrichuv Hradec, Czechia) and potassium polyacrylate (Falconry, Kozmice, Czechia). The final NHA composition consisted of 86wt.% starch- glycerol mixture (43wt.% glycerol), 7 wt% potassium polyacrylate, and 7 wt% zeolite. NHA was prepared by thermoplasticization at 140 °C by passing one cycle in a hot-melt extruder using citric acid as a crosslinking agent. The synthetic superabsorber polymer (SAP) treatment consisted of 100% potassium polyacrylate. Details on NHA preparation and nitrogen release characteristics are described by Skarpa et al.^22^.

The nutrient source was NPKS fertilizer (YARA Mila Complex 12-11-18-8; YARA Agri Czech Republic, Prague, Czechia). The hydrogels, fertilizer and fertilizer-enriched hydrogels were applied in two dosage levels (I and II). The ratio of hydrogel to fertilizer was adjusted to ensure equal application rates of nitrogen (24 and 48 kg/ha) and hydrogel (15 and 30 kg/ha) in both dosage levels (Table 2).

**Table 2:** Treatment design and application rates.

Treatment	Dose of nutrients (kg/ha)				Hydrogel dose (kg/ha)
	*N*	*P*_2_O_5_	K_2_O	S	
Control	0	0	0	0	0
NHA I	0	0	0	0	15
SAP I	0	0	0	0	15
NHA-NPKS I	24	22	36	16	15
SAP-NPKS I	24	22	36	16	15
NPKS I	24	22	36	16	0
NHA II	0	0	0	0	30
SAP II	0	0	0	0	30
NHA-NPKS II	48	44	72	32	30
SAP-NPKS II	48	44	72	32	30
NPKS II	48	44	72	32	0

The blue-seed poppy variety “MS Harlekyn” (National Agricultural and Food Center, Luzianky, Slovak Republic) was used as a model crop. The experiment followed a Randomized Complete Block Design. Each treatment was replicated 4 times (4 plots per treatment) each year (experimental section). The area of each plot was 15 m^2^ (10 × 1,5 m). The allocation of replicates across the area of each experimental section was consistent for all years (Figure S1).

Fertilizers and hydrogels were manually applied to individual plots one day before sowing and incorporated into the soil immediately after application. Sowing took place on 28 February 2022, 1 March 2023, and 19 March 2024.

Harvesting was carried out after physiological maturity (27 July 2022, 20 July 2023, and 22 July 2024) using a Haldrup C-85 plot harvester (Haldrup GmbH, Ilshofen, Germany). Seed yield was measured using a digital scale (KERN DS 60K0.2, KERN and Sohn GmbH, Germany). Grain moisture content was determined with a portable grain moisture meter Wile 78 Crusher (Farmcomp Oy, Tuusula, Finland) and yield was standardized to 8.0% moisture and expressed in tons per hectare (t/ha). The 1000-seeds weight was determined using a scale KERN ARJ 220-4 M, KERN and Sohn GmbH (Balingen, Germany).

The agronomic efficiency (AE)^37^ was expressed for the fertilized treatments as the increase in seed yield (kg) per unit of nitrogen applied (kg) according to the Eq. 1) and per unit of hydrogel applied (kg) according to the Eq. 2):

1 $$\:{\mathrm{AE}}_{\mathrm{N}}\mathrm{(kg/kg)=}\frac{{\mathrm{Y}}_{\mathrm{FERT}}\mathrm{(t/ha)}\:-\:{\mathrm{Y}}_{\mathrm{CONTROL}}\mathrm{(t/ha)}}{{\mathrm{N}}_{\mathrm{DOSE}}\mathrm{(kg/ha)}}\times\mathrm{1000}$$

2 $$\:{\mathrm{AE}}_{\mathrm{H}}\mathrm{(kg/kg)=}\frac{{\mathrm{Y}}_{\mathrm{FERT}}\mathrm{(t/ha)}\:-\:{\mathrm{Y}}_{\mathrm{CONTROL}}\mathrm{(t/ha)}}{{\mathrm{Hydrogel}}_{\mathrm{DOSE}}\mathrm{(kg/ha)}}\times \mathrm{1000}$$

where AE_N_ is agronomic efficiency of nitrogen, AE_H_ is agronomic efficiency of hydrogel, Y_FERT_ is fertilized treatment yield of poppy seed, Y_CONTROL_ is unfertilized treatment yield of poppy seed, N_DOSE_ is nitrogen dose applied by fertilizer, and Hydrogel_DOSE_ is hydrogel dose applied.

Soil sampling (0–15 cm depth) was carried out after poppy each year. Fresh fine samples were stored at 4 °C and analyzed for dehydrogenase activity (DHA) using 2,3,5-triphenyltetrazolium chloride (TTC) method^38^, and basal respiration (BR) using MicroResp^®^ device (The James Hutton Institute, Scotland) according to Campbell et al.^39^.

### Economic analysis

A partial budget analysis^40^ was performed to assess the cost effectiveness of hydrogels treatments on poppy seed production. This procedure considers only major differences between treatments (fertilization) without considering all costs and benefits. Therefore, only the cost of fertilizer or/and hydrogels, their applications (12 € for each treatment) and price of poppy seed were considered. All non-fertility costs (e.g., seed costs, field operations, plant protection) were held constant across treatments and were not included in the calculation. The cost of 1 ton of used fertilizer (YARA Mila Complex) was 840 €; the cost of 1 ton of SAP and NHA hydrogels was 12 000 and 5 400 € respectively. The prices of fertilizers for treatments were recalculated according to the corresponding applied doses (Table 2). The price of harvested commodity (culinary poppy seed) was 2400 €/t. The prices were based on the actual market values at the end of 2024.

However, the inherent volatility input (fertilizer/hydrogels) and output (seed of poppy) prices represents a challenge for accurate economic analysis. This approach focuses on practical economic variability; therefore, scenario-based sensitivity analysis was preferred over statistical confidence intervals, as it more realistically represents uncertainty arising from market and climatic fluctuations. Therefore, additional sensitivity analysis^41^ was performed under three different scenarios to accommodate possible market dynamics and to assess the effects of input and output price changes on the compared treatments of fertilization. These scenarios were: (Sc. 1) increase cost of hydrogels/fertilizer by 10% but fixed commodity price, (Sc. 2) increase commodity price by 10% with fixed hydrogels/fertilizer cost and (Sc. 3) increase cost of hydrogels/fertilizer by 10% and decrease commodity price by 10% (worst case scenario from farmers’ perspective). The average yield of poppy over the three years of the experiment was used for the economic evaluation. Confidence intervals for average yields were not included, as they would represent within-year experimental variability rather than the economic uncertainty captured by the scenario-based sensitivity analysis, which better reflects market and climatic risks influencing profitability.

### Statistical analysis

The effect of the hydrogels, fertilizer and their mixtures were assessed using ANOVA. Before performing ANOVA, the assumptions of normality and homogeneity of variances were tested using the Shapiro–Wilk and Levene’s tests, respectively. ANOVA was used to evaluate the effects of hydrogel type, dose, fertilizer, and their combinations. Two model structures were used:

(i) Per-year analyses, performed as a one-way ANOVA with Treatment as a fixed factor and Plot as a random factor (yield, 1000 seed weight, AE_N_, AE_H_, DHA, and BR), and.
(ii) Combined analyses, performed as a mixed two-way ANOVA with Year and Treatment as fixed factors and Plot (Year) as a random factor.

When appropriate, a factorial ANOVA including the main effects of Hydrogel type and Dose, and their interaction (Type × Dose), was used to partition the total variability. For each model, the F-statistic, degrees of freedom (df), and p-values for main effects and their interactions were calculated and are reported in Supplementary Tables S1–S3. The effect sizes were expressed as eta-squared (η² = SSeffect/SStotal) and partial eta-squared (partial η² = SSeffect/(SSeffect + SSerror)), representing the proportion of total variance explained by each factor. After a significant omnibus F-test (*p* ≤ 0.05), Fisher’s Least Significant Difference (LSD) test was applied for post-hoc multiple comparisons among treatment means. All statistical analyses were conducted using Statistica 14 CZ software^42^. Results are expressed as means ± standard deviations (SD) or standard errors (SE), as appropriate.

## Results

### Seed yield of poppy and agronomic efficiency

**Fig. 2:**
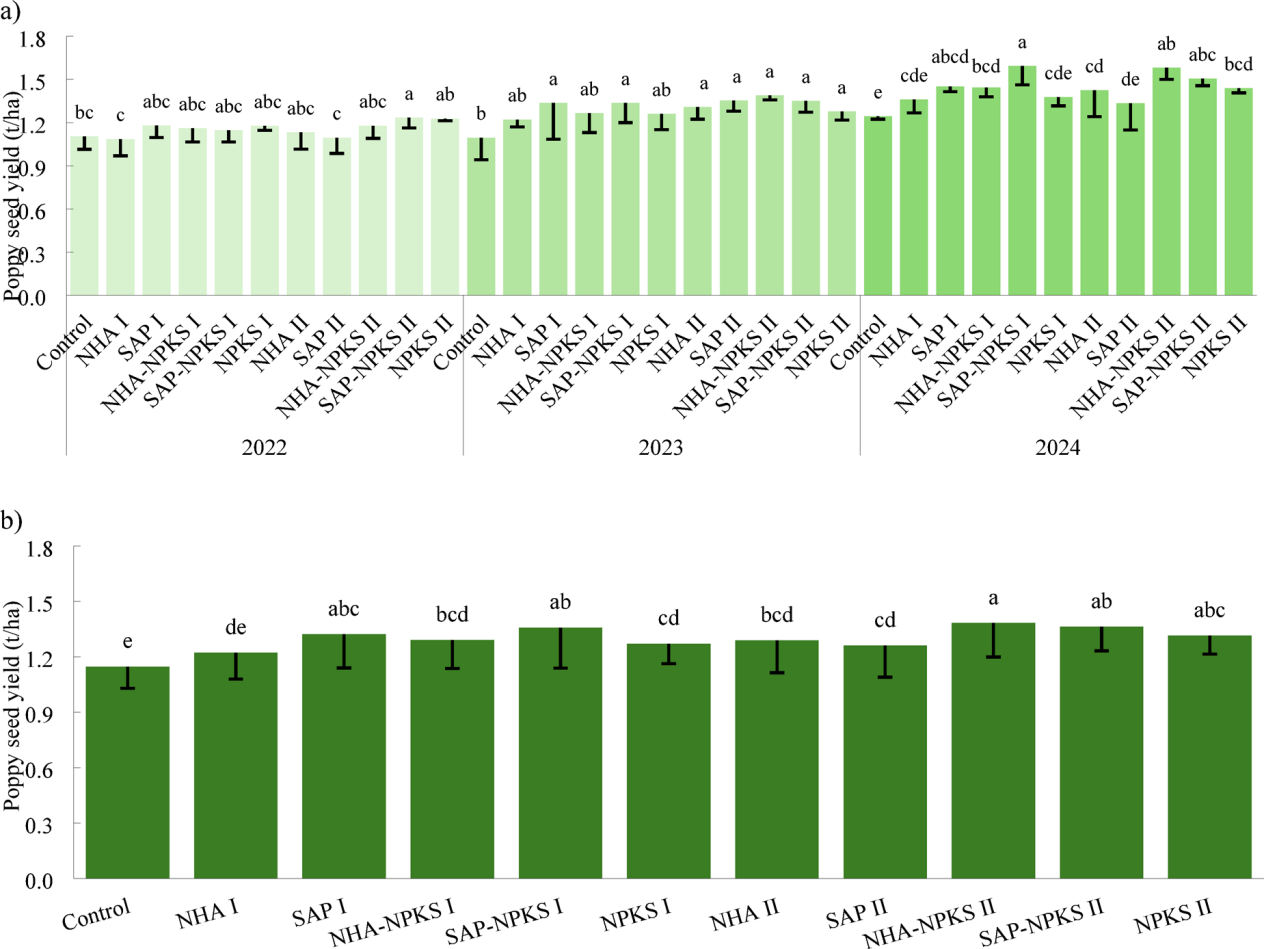
Effects of fertilization on poppy seed yield (t/ha) in 2022, 2023, 2024 (**a**), and average of three years (**b**). Control: treatment without fertilization; NHA: fertilized with bio- natural-based hydrogel; SAP: fertilized with synthetic hydrogel; NPKS: fertilized with NPKS fertilizer; NHA-NPKS: fertilized with NPKS fertilizer-enriched bio- natural-based hydrogel; SAP-NPKS: fertilized with NPKS fertilizer-enriched synthetic hydrogels. Roman numerals I and II indicate hydrogels/fertilizer rates. The columns marked by different lower-case letters indicate significant differences among treatments (each year was evaluated separately). The columns represent the arithmetic means (*n = 4*), standard deviation is expressed by error bars. The values *F*, *df*, and *P* for main effects and interactions are given in Tables S1.

The effects of hydrogels (SAP, NHA) enriched and not-enriched with fertilizer applied at two doses (I and II) on poppy seed yield are presented in Fig. 2. In all years, it is evident that the higher nutrient dose (II) applied in conventional fertilizer (NPKS) relatively increased seed yield in comparison with the lower dose (NPKS I) by 1.6% (2023), 4.7% (2022) and 5% (2024). The increase in yield of poppy seed caused by the higher NPKS dose was significant compared to the control in 2023 and 2024.

The yield response was also influenced by hydrogel dose, which accounted for 32.3% (η² = 0.323, Partial η² = 0.487, *p* = 0.335) of total seed yield variability, while hydrogel type explained 7.6% (η² = 0.076, Partial η² = 0.183, *p* = 0.638). A higher dose of synthetic SAP relatively reduced seed production (by 5.3% on average), whereas a higher dose of a natural-based hydrogel increased poppy seed yield: Control (1.15 t/ha, 100%) ˂ NHA I (1.22 t/ha, 106.5%) ˂ NHA II (1.29 t/ha, 112.3%).

The highest seed yields were observed in all years with fertilizer-enriched hydrogels. At the lower fertilizer rate (I), except in 2022, the combination of fertilizer with synthetic SAP had a higher effect on production compared to NHA (Fig. 2). This corresponded to the effect of pure SAP. At the higher nutrient rate (II), a higher increase in poppy yield for the fertilizer-NHA combination was observed (in 2023 and 2024). The effect of soil application of the tested fertilizers on seed yield, expressed as a mean for lower dose of hydrogels/fertilizer (I), was as follows: 1.15 ± 0.12 t/ha (Control) ˂ 1.22 ± 0.14 t/ha (NHA) ˂ 1.27 ± 0.11 t/ha (NPKS) ˂ 1.29 ± 0.15 t/ha (NHA-NPKS) ˂ 1.32 ± 0.18 t/ha (SAP) ˂ 1.36 ± 0.22 t/ha (SAP-NPKS). In contrast, when higher rates (II) were used, the effect of the tested fertilizer types was as follows: 1.15 ± 0.12 t/ha (Control) ˂ 1.26 ± 0.17 t/ha (SAP) ˂ 1.29 ± 0.17 t/ha (NHA) ˂ 1.32 ± 0.10 t/ha (NPKS) ˂ 1.36 ± 0.13 t/ha (SAP-NPKS) ˂ 1.38 ± 0.18 t/ha (NHA-NPKS).

The 1000-seed weight was not significantly affected by fertilization in 2022 and 2023 (Table 3). In 2024, the significantly highest poppy seed weight was found in the higher fertilizer rate (II) treatments as follows: NHA-NPKS ˂ NPKS ˂ SAP-NPKS. Consistent with the results of the 3rd year of testing, the relatively highest average seed weight was found on the treatments fertilized with higher rates of pure fertilizer and hydrogels enriched by fertilizer. Their increased doses (II) resulted in an increase of poppy seed weight compared to the lower doses (I), by 6.6% (NPKS), 7.2% (NHA-NPKS) and 11.4% (SAP-NPKS), respectively. Pure hydrogels did not affect seed weight significantly.

**Table 3:** Effects of fertilization on 1000 seed weight (g).

Treatment	2022	2023	2024	Average
Control	0.44^a^ ± 0.03	0.37^a^ ± 0.03	0.42^de^ ± 0.01	0.41^d^ ± 0.04
NHA I	0.43^a^ ± 0.01	0.39^a^ ± 0.02	0.46^bcd^ ± 0.03	0.43^bcd^ ± 0.04
SAP I	0.44^a^ ± 0.03	0.38^a^ ± 0.04	0.45^cde^ ± 0.05	0.42^cd^ ± 0.05
NHA-NPKS I	0.43^a^ ± 0.01	0.40^a^ ± 0.05	0.43^cde^ ± 0.03	0.42^d^ ± 0.03
SAP-NPKS I	0.44^a^ ± 0.02	0.37^a^ ± 0.01	0.43^cde^ ± 0.06	0.41^d^ ± 0.05
NPKS I	0.45^a^ ± 0.01	0.39^a^ ± 0.03	0.43^cde^ ± 0.02	0.42^cd^ ± 0.03
NHA II	0.44^a^ ± 0.01	0.38^a^ ± 0.01	0.41^e^ ± 0.03	0.41^d^ ± 0.03
SAP II	0.45^a^ ± 0.01	0.37^a^ ± 0.02	0.47^bc^ ± 0.10	0.43^bcd^ ± 0.07
NHA-NPKS II	0.45^a^ ± 0.03	0.39^a^ ± 0.01	0.50^ab^ ± 0.03	0.45^abc^ ± 0.05
SAP-NPKS II	0.46^a^ ± 0.02	0.39^a^ ± 0.03	0.54^a^ ± 0.05	0.46^a^ ± 0.07
NPKS II	0.44^a^ ± 0.02	0.39^a^ ± 0.04	0.52^a^ ± 0.02	0.45^ab^ ± 0.06

Roman numerals I and II indicate hydrogels/fertilizer rates. The values are expressed as the arithmetic mean (*n = 4*) ± standard deviation. Different lower-case letters indicate significant differences among treatments (each year was evaluated separately). The values *F*, *df*, and *P* for main effects and interactions are given in Tables S1.

The agronomic efficiency of nitrogen (AE_N_) and hydrogel (AE_H_) is shown in Table 4.

The lower rate of nitrogen applied by NPKS fertilizer resulted in significantly higher AE_N_ compared to the higher rate in the average of three years (Table 4). The largest difference in AE_N_ between nitrogen rates was observed in 2023, where 1 kg of nitrogen applied at the lower rate increased poppy seed yield by 6.9 kg, while the yield increase at the higher rate was 3.8 kg of seed.

The agronomic nitrogen efficiency of fertilizer-enriched hydrogels applied at a lower rate was significantly higher when using synthetic SAP. Significant increase in AE_N_ for SAP-NPKS I compared to NPKS I was found in 2023 (+ 46.4%) and 2024 (+ 160.7%), averaging 69.2% over the three years (Table 4). An average increase in AE_N_ (+ 15.4%) was also observed with the use of fertilizer-enriched NHA (NHA-NPKS I), but not significant. Higher rates of hydrogels enriched by fertilizer did not statistically affect the efficiency of applied nitrogen. The relatively highest AE_N_ value was found for the NHA-NPKS II treatment (4.9; 100%), followed by SAP-NPKS II (91.8%) and NPKS II (71.4%). The total variability of AE_N_ was significantly influenced mainly by the dose of hydrogel (η² = 0.570, Partial η² = 0.851, *p* = 0.021) while the type of hydrogel explained 11.7% (η² = 0.117, Partial η² = 0.539, *p* = 0.286).

With pure hydrogel applied at a lower rate, poppy seed yield increased significantly with synthetic SAP. The average AE_H_ for SAP I was 11.7 (i.e. the seed yield increased by 11.7 kg due to the application of 1 kg of SAP). The agronomic efficiency of the synthetic hydrogel was more than twice as high compared to NHA (Table 4). In contrast, for the higher dose of hydrogel, the efficiency of NHA was similar to the effect of its lower dose, whereas in the case of SAP, AE_H_ was significantly reduced (more than threefold decrease).

The effect of hydrogels (AE_H_) on poppy yield increased when used in combination with fertilizers. In the case of natural hydrogel enriched by fertilizer (NHA-NPKS) compared to its pure form (NHA), a significant increase in AE_H_ was observed for both doses (I + 92%, II + 68.1%). In the case of synthetic hydrogel, an increase in AE_H_ was also observed between the SAP-NPKS and SAP, but significantly only for the higher dose (+ 89.5%).

**Table 4:** Effects of fertilization on agronomic efficiency of nitrogen (AE_N_), and agronomic efficiency of hydrogel (AE_H_).

Treatment	AE_*N*_ (kg)				AE_H_ (kg)			
	2022	2023	2024	Average	2022	2023	2024	Average
NHA I	Nc	Nc	Nc	Nc	−1.2^c^ ± 1.1	8.4^b^ ± 3.5	7.8^cd^ ± 2.5	5.0^cde^ ± 1.9
SAP I	Nc	Nc	Nc	Nc	5.1^a^ ± 0.4	16.1^a^ ± 3.3	13.8^b^ ± 0.8	11.7^ab^ ± 1.8
NHA-NPKS I	2.4^a^ ± 0.4	7.1^b^ ± 0.8	8.4^b^ ± 1.0	6.0^b^ ± 0.9	3.9^abc^ ± 0.6	11.4^ab^ ± 1.3	13.4^b^ ± 1.6	9.6^b^ ± 1.4
SAP-NPKS I	1.8^a^ ± 0.4	10.1^a^ ± 1.5	14.6^a^ ± 2.3	8.8^a^ ± 1.8	2.9^abc^ ± 0.6	16.1^a^ ± 2.4	23.3^a^ ± 3.7	14.1^a^ ± 2.9
NPKS I	3.1^a^ ± 1.2	6.9^b^ ± 1.3	5.6^bcd^ ± 0.9	5.2^b^ ± 0.8	Nc	Nc	Nc	Nc
NHA II	Nc	Nc	Nc	Nc	1.0^abc^ ± 0.5	7.1^b^ ± 1.3	6.0^cd^ ± 2.7	4.7^de^ ± 1.2
SAP II	Nc	Nc	Nc	Nc	−0.3^bc^ ± 0.7	8.6^b^ ± 1.5	3.1^d^ ± 2.8	3.8^e^ ± 1.5
NHA-NPKS II	1.6^a^ ± 0.3	6.1^bc^ ± 1.3	7.1^bc^ ± 0.7	4.9^bc^ ± 0.9	2.5^abc^ ± 0.4	9.8^b^ ± 2.1	11.3^bc^ ± 1.1	7.9^bc^ ± 1.4
SAP-NPKS II	2.7^a^ ± 0.3	5.3^bc^ ± 1.0	5.5^cd^ ± 0.3	4.5^bc^ ± 0.5	4.4^ab^ ± 0.4	8.6^b^ ± 1.6	8.7^bc^ ± 0.5	7.2^bcd^ ± 0.8
NPKS II	2.6^a^ ± 0.8	3.8^c^ ± 1.0	4.1^d^ ± 0.2	3.5^c^ ± 0.4	Nc	Nc	Nc	Nc

Roman numerals I and II indicate hydrogels/fertilizer rates. The values are expressed as the arithmetic mean (*n = 4*) ± standard error. Different lower-case letters indicate significant differences among treatments (each year was evaluated separately). Nc – no calculation. The values *F*, *df*, and *P* for main effects and interactions are given in Tables S2.

### Soil microbial activity and biomass

No significant effects of any treatment of hydrogels (SAP, NHA) applied either solely or with NPS was observed on soil dehydrogenase activity (DHA) in 2022 (Table 5). In the next year 2023, all types of amendments (except of NHA I) increased DHA in comparison to Control value, and increased value in SAP I, which was even higher in combination with higher NPKS (SAP-NPKS II) as well as in all treatments with higher or/and combined NHA amendment (NHA-NPKS I, NHA II, NHA-NPKS II). Moreover, NHA II enhanced soil DHA significantly more than both doses of NPKS (I and II), showing the highest enzyme values in 2023, 2024 and in 3-year average (Table 5). In 2024, only treatments with sole NHA (I, II), NHA-NPKS I, and NPKS I were increased over Control, while SAP II was decreased. In 3-year average, SAP applied solely (in low dose I) or combined (I, II) increased DHA over Control values but not compared to NPKS (I, II). Only NHA-NPKS I and NHA II enhanced DHA more than amendment of fertilizers.

**Table 5:** Effects of fertilization on dehydrogenase activity (DHA) of microbial biomass and basal soil respiration (BR).

Treatment	DHA (µg TPF/g/h)				BR (µg CO_2_/g/h)			
	2022	2023	2024	Average	2022	2023	2024	Average
Control	5.7^ab^ ± 0.5	5.0^d^ ± 0.3	8.2^d^ ± 2.1	6.3^e^ ± 1.8	0.34^a^ ± 0.06	0.36^a^ ± 0.07	0.57^cd^ ± 0.12	0.43^ab^ ± 0.14
NHA I	5.5^ab^ ± 0.5	5.3^d^ ± 0.9	9.1^b^ ± 0.7	6.6^cde^ ± 1.9	0.30^ab^ ± 0.04	0.30^a^ ± 0.03	0.57^cde^ ± 0.11	0.39^bcde^ ± 0.14
SAP I	5.8^a^ ± 1.2	6.5^c^ ± 0.4	8.3^d^ ± 0.5	6.8^c^ ± 1.3	0.28^ab^ ± 0.02	0.30^a^ ± 0.05	0.53^def^ ± 0.16	0.37^de^ ± 0.15
NHA-NPKS I	5.7^ab^ ± 0.7	7.1 ^ab^ ± 0.8	9.0^bc^ ± 1.0	7.2^b^ ± 1.6	0.28^ab^ ± 0.03	0.33^a^ ± 0.05	0.54^def^ ± 0.08	0.38^cde^ ± 0.13
SAP-NPKS I	5.4^ab^ ± 0.4	6.9^abc^ ± 0.7	8.4^d^ ± 1.3	6.9^c^ ± 1.5	0.27^abc^ ± 0.02	0.30^a^ ± 0.05	0.61^bcd^ ± 0.18	0.39^bcde^ ± 0.19
NPKS I	5.6^ab^ ± 0.8	6.7^bc^ ± 0.5	8.4^cb^ ± 0.8	6.9^c^ ± 1.4	0.34^a^ ± 0.08	0.22^b^ ± 0.03	0.66^ab^ ± 0.21	0.41^abcd^ ± 0.23
NHA II	5.5^ab^ ± 0.7	7.3^a^ ± 0.7	10.0^a^ ± 1.5	7.6^a^ ± 2.1	0.32^ab^ ± 0.10	0.33^a^ ± 0.06	0.70^a^ ± 0.31	0.45^a^ ± 0.26
SAP II	5.3^ab^ ± 0.6	6.9^abc^ ± 0.9	7.3^e^ ± 1.1	6.5^de^ ± 1.2	0.35^a^ ± 0.10	0.29^ab^ ± 0.04	0.64^abc^ ± 0.13	0.43^abc^ ± 0.18
NHA-NPKS II	5.2^ab^ ± 0.6	7.1^ab^ ± 0.3	8.1^d^ ± 0.7	6.8^c^ ± 1.3	0.25^bc^ ± 0.07	0.29^ab^ ± 0.06	0.59^bcd^ ± 0.19	0.38^de^ ± 0.19
SAP-NPKS II	5.1^b^ ± 0.5	7.1^ab^ ± 0.6	8.3^d^ ± 0.9	6.8^c^ ± 1.5	0.33^ab^ ± 0.05	0.36^a^ ± 0.10	0.46^f^ ± 0.11	0.38^bcde^ ± 0.11
NPKS II	5.3^ab^ ± 0.3	6.7^bc^ ± 0.5	8.0^d^ ± 0.8	6.7^cd^ ± 1.2	0.20^c^ ± 0.09	0.36^a^ ± 0.08	0.50^ef^ ± 0.12	0.35^e^ ± 0.16

Roman numerals I and II indicate hydrogels/fertilizer rates. The values are expressed as the arithmetic mean (*n = 16*) ± standard error. Different lower-case letters indicate significant differences among treatments (each year was evaluated separately). The values *F*, *df*, and *P* for main effects and interactions are given in Tables S3.

In 2022, soil basal respiration (BR) was decreased compared to Control in treatments with high fertilizer dose (NPKS II), applied solely or combined with NHA (Table 5). In 2023, only NPKS I (low dose) exerted negative effect on BR. In 2024, NPKS II again solely or combined (SAP-NPKS II) showed significant decrease in BR, while NPKS I and NHA II increased the values over Control. In 3-year average, mainly insignificantly different or negative effects of tested amendments on BR were found, the strongest BR reduction was derived by NPKS II, SAP I, NHA-NPKS I and II (both; Table 5).

### Economic evaluation of poppy production

Table 6 describes the results of the economic evaluation of poppy production for the tested fertilization treatments under different price scenarios, considering the variable price of fertilizers and commodity (poppy seed). At lower fertilizer doses (I), the application of pure SAP (SAP I) and SAP enriched by fertilizer (SAP-NPKS I) was the most profitable in each scenario. The net profit of the NHA I treatment was approximately 38% of the SAP I profit. In the case of fertilizer-enriched hydrogels, the difference between net profit of SAP and NHA was lower (Table 6).

Higher doses of hydrogels and their fertilizer-enriched forms increased the cost of poppy production and reduced profits. The use of higher doses of SAP (SAP II, SAP-NPKS II) was unprofitable (loss-making in all scenarios). The higher poppy yield obtained after applying natural hydrogels at a higher dose (II) increased the profit, in the case of using pure NHA in the range of 115–199 €/ha, when using NHA-NPKS depending on the type of scenario, as shown in Table 6 (highest profit in Sc. 2, conversely, loss in the case of Sc. 3).

**Table 6:** Economic evaluation of poppy fertilization.

Treatment	Fertilization cost (€/ha) *			Total revenue (€/ha) **	Revenue increase (€/ha) ***	Net profit (€/ha) ****			
	Fert.	Hydr.	Total						
						Sc. 2024	Sc. 1	Sc. 2	Sc. 3
Control	0	0	0	2 754	-	-	-	-	-
NHA I	0	81	93	2 934	180	87	79 (−8)	105 (+ 18)	61 (−26)
SAP I	0	180	192	3 173	419	227	209 (−18)	269 (+ 42)	167(−60)
NHA-NPKS I	168	81	261	3 097	343	82	57 (−25)	117 (+ 35)	23 (−59)
SAP-NPKS I	168	180	360	3 261	507	147	112 (−35)	198 (+ 51)	61(−85)
NPKS I	168	0	180	3 051	297	117	100 (−17)	147 (+ 30)	71(−46)
NHA II	0	162	174	3 093	339	165	149 (−16)	199 (+ 34)	115 (−50)
SAP II	0	360	372	3 028	274	−98	−134 (−36)	−70 (+ 28)	−161 (−63)
NHA-NPKS II	336	162	510	3 321	567	57	8 (−50)	114 (+ 57)	−49(−106)
SAP-NPKS II	336	360	708	3 273	519	−189	−259 (−70)	−137 (+ 52)	−310 (−121)
NPKS II	336	0	348	3 156	402	54	21 (−34)	95 (+ 41)	−19 (−74)

*Fertilization cost (Total) = Price of fertilizer (Fert.) + Price of hydrogel (Hydr.) + Application cost (12 €/ha). **Total revenue (€/ha) = Average yield (t/ha) Purchase price (1 t of poppy seed). ***Revenue increase (€/ha) = Total revenue of fertilized treatments (€/ha) – Total revenue of Control treatment (€/ha). ****Net profit (€/ha) = Revenue increase (€/ha) - Fertilization cost (Total) (€/ha). Scenario: Sc. 2024: actual prices at the end of the year 2024; Sc. 1: increase cost of hydrogels/fertilizer by 10% but fixed commodity price; Sc. 2: increase commodity price by 10% with fixed hydrogels/fertilizer cost; Sc. 3: increase cost of hydrogels/fertilizer by 10% and decrease commodity price by 10%. The net profit for Scenarios 1–3 is expressed as an increase (+ €/ha) or decrease (- €/ha) in profit compared to the Scenario 2024.

## Discussion

The effect of the tested types of hydrogels on poppy (*Papaver somniferum* L.) seed yield varied depending on the type and application rate. At a lower dose, the synthetic SAP increased seed yield more effectively compared to the natural hydrogel (NHA), whereas the opposite trend was observed at the higher dose. This pattern was consistent for both pure hydrogels and their fertilizer-enriched formulations.

Specifically, the lower application rate of pure SAP and SAP combined with fertilizer (SAP-NPKS I) significantly improved poppy seed yield. These results align with prior studies that demonstrated increased crop yields following SAP application due to their high water absorption and retention capacity^43^, as well as improvements in soil physical and chemical properties^44,45^. Additionally, SAPs have been shown to reduce nutrient losses^46,47^, and enhance fertilizer use efficiency^45^. In the present study, the agronomic efficiency of nitrogen (AE_N_) was significantly higher in the SAP-NPKS treatment compared to NPKS alone (Table 4).

Yield differences following hydrogel application are attributed to the feedstock’s composition, hydrogel formulation and method of application^48^. Synthetic SAP generally show stronger positive effects on yield than natural hydrogels^9^. Zheng et al.^9^ reported an average 12.8% in crop yield from SAP application (95% confidence intervals: 12.1–13.4%, *p* < 0.01). In our trial, pure SAP application increased poppy yield by 12.4% o average across both doses, whereas pure NHA increased yield by 9.4%. Zheng et al.^9^ also found a 15.2% increase in oilseed-yields, including poppy, after SAP application, although data specific to poppy remain scarce. One of the few relevant studies showed improved oilseed rape yield under drought and irrigation conditions with the application of anionic cellulose-based polyacrylate hydrogel at 40 kg/ha^49^.

However, high doses of synthetic SAPs may negatively affect plant growth. Situ et al.^34^ found that excessive use of synthetic SAP reduced biomass and roots and stems–leaves, likely due to ion imbalances (elevated K^+^ and Na^+^, reduced Ca^2+^ and Mg^2+^). This may explain observed reductions in germination rate, plant height and yield. In our study the SAP II treatment led to a relative yield decline in two of the three trial years. On average, the SAP II treatment yielded 5.6% less than the NHA II treatment.

The effect of hydrogels on seed weight and poppy production also depended on weather conditions during vegetation periods. In particular, in relatively dry and cool years (2022 and 2023) seed weight was not significantly affected by fertilization (Table 3). On the contrary, 2024 growing period was characteristic of sufficient water supply and higher temperatures, which supported soil microbial activity (as reflected in DHA and BR, Table 5), in particular in the presence of bio-SAP-based fertilizers. The elevated microbial activity increases soil organic matter turnover and contribute to soil fertility^50^, with a direct impact on crop yields^51,52^, as observed in this work.

However, at higher SAP rates the reduced growth was observed, which may stem (apart from ion imbalance), from the presence of acrylic acid. Chen et al.^53^ reported damage to the organizational structure and cellular morphology of maize roots and the membrane system of root cells. Puoci et al.^54^ described acrylate hydrogel intermediates as cytotoxic. Additionally, excessive SAP can compete with plants for water under drought conditions, potentially exacerbating water stress in plants and thereby reducing yields^55,56^. This aligns with Zheng et al.^9^ who concluded that SAP rates > 90 kg/ha do not significantly improve yields and may even be detrimental, while the application rate 45–90 kg/ha exhibited positive results.

Although recent research focuses increasingly on natural polymers or organic-inorganic hybrid compounds, most studies still involve synthetic SAPs (acrylate and acrylamide-based)^9^. Nevertheless, several studies confirm yield benefits from bio-based hydrogels^22,57–60^, which offer key advances such as biocompatibility, non-toxicity, and biodegradability. However, “biodegradability” is a broad term and does not necessarily reflect degradation rates. The NHA used in this study, composed primarily of potato starch (86 wt%), is highly biodegradable. Therefore, NHA was superior in enhancing microbial activity as starch degradation products provide carbon source for microorganisms. Based on CO_2_ released during analysis, Guo et al.^61^ reported that 78.34% of starch degraded within 14 days. The starch decomposition rate reported in laboratory tests is difficult to achieve under field conditions. Starch and starch based polyurethane materials (starch-polyhydroxyurethanes) lost about 44.1 and 66.4% of their weight, respectively, after 60 days burial in soil^62^. The biodegradability of NHA was also supported by elevated dehydrogenase activity and basal respiration measured in soil after harvest (Table 5). Soil microbial activity was more strongly stimulated by NHA and NHA-NPKS than by NPKS alone, likely due to excellent biodegradability of starch^63^ and glycerol^64^.

While SAP also stimulated soil microbiome which is reflected in elevated DHA^65^, the stimulation was weaker, likely due to slow biodegradation of polyacrylic gels that are primarily degraded fungi such as *Phanerochaete chrysosporium*^66^. Although NHA-NPKS enhanced DHA, it did not significantly increase basal respiration over the three-year average. This is likely due to the limited stimulator effect of NPKS amendment, in agreement with study of Kulachkova et al.^67^, who reported minimal BR after Nitroammofoska-1 application (NPKS 21-10-10-2, similar to the composition of YARA Mila Complex 12-11-18-8) to urban lawn soil.

On average, BR was higher following NHA application compared to SAP, by 4.6% at the lower dose, and 5.0% at the higher dose. The poor biodegradability of synthetic SAPs raises environmental concerns^68^. Their degradation rate is typically 0.45 to 0.82% over 24 weeks depending on soil type but not on soil temperature. Detailed study showed that the polyacrylate superabsorbent main chain degraded in the soils at rates of 0.12–0.24% per 6 months^11^. Aging via chemical, photolytic, and mechanical stress can lead to fragmentation and formation of microplastic particles, which may leach into deeper soil layers or into adjacent ecosystems, potentially impacting microbial communities and plant growth^69,70^.

Dehydrogenase activity is a well-established indicator of overall microbial activity^71^. Significantly higher DHA values were observed found in two of the three years and on average in the NHA II treatment (Table 4). Soil treated with NHA and NHA-NPKS exhibited the highest DHA (7.1 a ± 1.8) significantly greater than SAP treatments (6.8 b ± 1.3) and non-hydrogel controls (6.6 c ± 1.5) (p˂0.05). Excessive doses of SAP (based on polyacrylic acid) have been reported to supress microbial respiration in sandy soils^72^. Soil microbial biomass plays a crucial role in nutrient cycling and natural based hydrogel may further support microbial growth by providing degradable organic substances^73^, enhancing microbial diversity^74^, and ultimately improving soil vitality, plant growth and survival rates^73^.

In addition to agronomic and environmental performance, economic viability is crucial for hydrogels adoption. Although economic analysis of hydrogel use remain limited, they are essential to evaluate practical constrains and inform farmers. Commercial synthetic SAPs based on polyacrylic acid are costly despite their high swelling capacity^68^. Natural hydrogels represent a lower-cost, faster-degrading alternatives with promising market potential^48^.

In this work, a lower dose of potassium polyacrylate (SAP I) resulted in the highest net profit up to 269 €/ha (Sc. 2, Table 5) due to the increase in poppy seed yield. The fertilizer-enriched SAP (SAP-NPKS I) was profitable compared to fertilizer (NPKS I) (average of all calculated scenarios: +20.8 €/ha). The increase in yield and net profit at a lower dose of NHA (NHA I) was also economically beneficial (61–105 €/ha). The use of NHA enriched with fertilizer (NHA-NPKS I) did not exhibit an increased profit compared to SAP. In this context, however, it is also important to consider the environmental compatibility of natural-based hydrogels, even though they may be less economically attractive.

These results suggest that poppy is among the crops for which hydrogel application can be economically justified. Yet, profitability depends on crop type. For example, despite yield increases, SAP costs were not offset by revenues in grain crops (net loss of 11 €/ha)^9^. On the other hand, net profit gains have been documented in maize^75^, sugarcane^76^, potatoes^44^, Indian mustard^77^, and summer pearl millet^78^. In our study high-dose NHA (NHA II) indicate an net profit increased by 115 to 199 €/ha, while high-dose SAP (SAP II) appeared to be economically unviable.

## Conclusion

This study demonstrates that the application of hydrogels, particularly when enriched with fertilizers, can significantly enhance the yield and nutrient-use efficiency of culinary poppy cultivated under drought-prone conditions. While low-dose synthetic SAP treatments provided the highest net economic returns, high-dose SAP applications proved less effective and potentially detrimental due to reduced biodegradability and possible phytotoxicity. In contrast, natural-based hydrogels (NHA), especially when combined with fertilizer, promoted soil microbial activity and showed consistent yield benefits at both application rates. Although the economic return from NHA was generally lower than from SAP, its environmental advantages, such as enhanced biodegradability and stimulation of beneficial soil microbiota, make it a compelling alternative for sustainable agriculture.

Overall, natural starch-based hydrogels enriched with fertilizer represent a viable, environmentally friendly strategy for improving soil water retention, nutrient efficiency, and crop performance in poppy cultivation. However, the composition of hydrogels (source of nutrients, e.g., potassium), site-specific conditions such as soil type, climate, and crop response variability must be considered when selecting the appropriate hydrogel type and dose for field application.

## Supplementary Information

Below is the link to the electronic supplementary material.

Supplementary Material 1

## Data Availability

The datasets generated and/or analysed during the current study are available from the corresponding author on reasonable request.

## Abbreviations

NHA: natural-based hydrogel
SAP: synthetic hydrogel
NPKS: mineral fertilizer YARA Mila Complex
NHA-NPKS: natural-based hydrogel enriched with fertilizer
SAP-NPKS: synthetic hydrogel enriched with fertilizer
AE_N_: agronomic efficiency of nitrogen fertilization
AE_H_: agronomic efficiency of hydrogel
DHA: dehydrogenase activity
BR: basal respiration
