# Supplementary material for: Comparative effects of synthetic and natural hydrogels enriched with fertilizer on poppy yield and soil health in drought-prone conditions
Source: Sci Rep. 2025 Dec 29;15:44694. doi: 10.1038/s41598-025-28213-0 (PMC12748965; doi:10.1038/s41598-025-28213-0)
Supplement: Supplementary file 1 — Supplementary Material 1 [file 41598_2025_28213_MOESM1_ESM.docx]

# **Comparative effects of synthetic and natural hydrogels enriched with fertilizer on poppy yield and soil health in drought-prone conditions**

Tomáš Kriška ^1^, Jiří Antošovský ^1^, Martin Brtnický ^1^, Jiří Kučerík ^1^, Jiří Holátko ^1^, Josef Jančár ^2^ and Petr Škarpa ^1,*^

^1^ Department of Agrochemistry, Soil Science, Microbiology and Plant Nutrition, Faculty of AgriSciences, Mendel University in Brno, Zemědělská 1, 61300 Brno, Czech Republic;

^2^ Institute of Materials Science, Faculty of Chemistry, Brno University of Technology, Purkyňova 118, 61200 Brno, Czech Republic

* Correspondence: [petr.skarpa@mendelu.cz](mailto:petr.skarpa@mendelu.cz); Tel.: +420 545 133 345


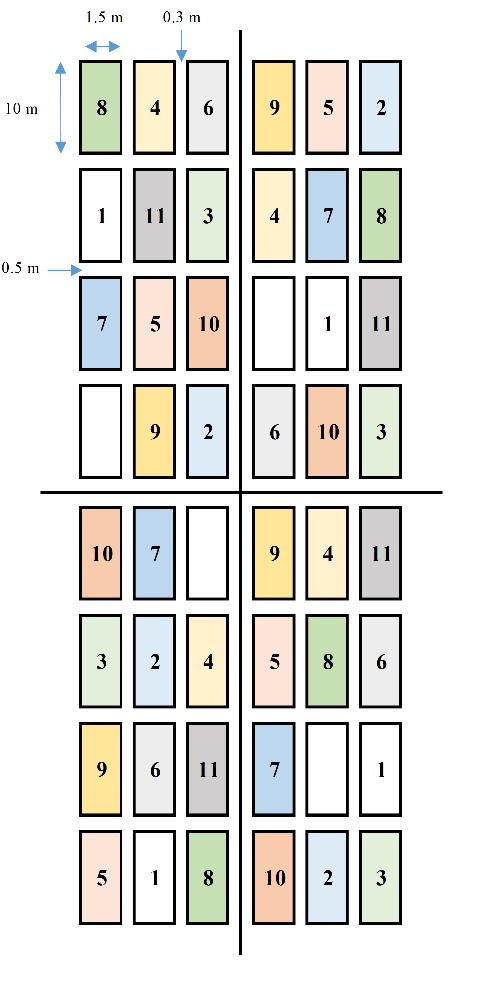


Figure S1. Treatment allocation on experimental section (2022-2024). Treatment designation: 1 Control; 2 NHA I; 3 SAP I; 4 NHA-NPKS I; 5 SAP-NPKS I; 6 NPKS I; 7 NHA II; 8 SAP II; 9 NHA-NPKS II; 10 SAP-NPKS II; 11 NPKS II.

Table S1. Analysis of variance: poppy yield (t/ha) and 1000 seed weight (g)

| Year | Source | poppy yield | | | 1000 seed weight | | |
| --- | --- | --- | --- | --- | --- | --- | --- |
|  |  | *df* | *F* | *P* | *df* | *F* | *P* |
| 2022 | Treatment | 10 | 1.31 | 0.265 | 10 | 0.69 | 0.730 |
| 2023 |  | 10 | 1.82 | 0.096 | 33 | 0.42 | 0.925 |
| 2024 |  | 10 | 4.22 | 0.001 | 43 | 3.94 | 0.001 |
| Average of three years | Year | 2 | 76.98 | 0.000 | 2 | 66.26 | 0.000 |
|  | \| Treatment \| \| --- \| | 10 | 5.15 | 0.000 | 10 | 3.38 | 0.001 |
|  | Year * treatment | 20 | 1.10 | 0.358 | 20 | 2.29 | 0.004 |

*df*: degree of freedom (n-1), *F*: F-ratio, *P*: p-value.

Table S2. Analysis of variance: agronomic efficiency of nitrogen (AE_N_) and hydrogel (AE_H_)

| Year | Source | AE_N_ (kg) | | | AE_H_ (kg) | | |
| --- | --- | --- | --- | --- | --- | --- | --- |
|  |  | *df* | *F* | *P* | *df* | *F* | *P* |
| 2022 | Treatment | 5 | 0.23 | 0.946 | 7 | 0.79 | 0.604 |
| 2023 |  | 5 | 1.18 | 0.357 | 7 | 0.82 | 0.578 |
| 2024 |  | 5 | 7.19 | 0.001 | 7 | 5.54 | 0.001 |
| Average of three years | Year | 2 | 43.1 | 0.000 | 2 | 55.21 | 0.000 |
|  | \| Treatment \| \| --- \| | 5 | 9.5 | 0.000 | 7 | 10.99 | 0.000 |
|  | Year * treatment | 10 | 4.2 | 0.000 | 14 | 2.37 | 0.009 |

*df*: degree of freedom (n-1), *F*: F-ratio, *P*: p-value.

Table S3. Analysis of variance: dehydrogenase activity (DHA) of microbial biomass and basal soil respiration (BR)

| Year | Source | DHA (μg TPF/g/h) | | | BR (μg CO_2_/g/h) | | |
| --- | --- | --- | --- | --- | --- | --- | --- |
|  |  | *df* | *F* | *P* | *df* | *F* | *P* |
| 2022 | Treatment | 10 | 1.60 | 0.111 | 10 | 7.53 | 0.000 |
| 2023 |  | 10 | 22.79 | 0.000 | 10 | 8.07 | 0.000 |
| 2024 |  | 10 | 6.18 | 0.000 | 10 | 2.77 | 0.003 |
| Average of three years | Year | 2 | 586.92 | 0.000 | 2 | 364.78 | 0.000 |
|  | \| Treatment \| \| --- \| | 10 | 8.58 | 0.000 | 10 | 3.19 | 0.001 |
|  | Year * treatment | 20 | 8.31 | 0.000 | 20 | 4.20 | 0.000 |

*df*: degree of freedom (n-1), *F*: F-ratio, *P*: p-value.
